# Occult HBV infection among Egyptian hepatocellular carcinoma patients

**DOI:** 10.1186/1743-422X-8-90

**Published:** 2011-03-03

**Authors:** Zeinab K Hassan, Mohamed M Hafez, Tarek M Mansor, Abdel Rahman N Zekri

**Affiliations:** 1 Virology and immunology Unit, Cancer Biology Department, National Cancer Institute, Cairo University, 1st Kasr El-Aini st, 11197 Cairo, Egypt; 2 Zoology Department, Faculty of Science, King Saud University, 2457 Riyadh 11451, Saudi Arabia; 3 Pharmacology Department, collage of pharmacy, King Saud University, 2457 Riyadh 11451, Saudi Arabia

## Abstract

**Background:**

Occult HBV infection accelerates the progression of liver fibrosis, cirrhosis, and finally leading to hepatocellular carcinoma (HCC). This study analyzed the occult HBV-genotypes in HCC patients.

**Methods:**

To achieve our objective, matched serum and tissue samples were collected from 40 HCC patients. Three sets of primers were used for the HBV-DNA detection by nested-PCR, which cover the HBV-genome; Core, Surface and X genes. Genotyping system based on PCR using type-specific primers was applied on HBV-DNA positive samples.

**Results:**

Intrahepatic occult HBV-DNA was detected in 62.5%, whereas; Serum occult HBV-DNA were detected in only 22.5% of HCC patients. In patients' positive for both anti-HBs and anti-HBc, 10% had occult HBV in serum. In serologically negative HCV patients, 63% had intrahepatic HBV-DNA, and 21% had HBV-DNA in serum samples. HBV-genotype D (32%) and B (24%) attributed predominantly to intrahepatic HBV infections in HCC patients, whereas HBV-genotype A (4%) and C (8%) infections were the least observed.

**Conclusion:**

This is the first study to show the genotypes of occult HBV infection in HCC Patients. We suggest that B or D may influence the outcome of HBV infection which may lead to the development of HCC.

## Introduction

Hepatocellular carcinoma (HCC) is one of the most common malignant tumors worldwide [1] ranging between 3% and 9% annually [2]. In Egypt, HCC reports to account for about 4.7% of chronic liver disease patients [3]. HBV and HCV infections are strongly associated with liver cirrhosis and HCC [4]. Africa is one of the highly endemic regions of HBV, with 5 genotypes A-E are reported as predominant genotypes in different countries [5,6]. HBV is a serious public health problem worldwide and major cause of chronic hepatitis, cirrhosis, and HCC [7,8]. The diagnosis of HBV infection is usually based on the detection of hepatitis B surface antigen (HBsAg). Occult hepatitis B is defined by the presence of HBV DNA in serum or liver in the absence of HBsAg [9,10]. HBV DNA can be detected in patients with chronic liver disease who are negative for HBsAg but positive for antibodies to hepatitis B core antigen (anti-HBc) [11,12]. The diagnosis depends on the sensitivity of HBV DNA assays and the prevalence of HBV infection in the population [11,13].

The occult HBV infection has frequently been identified in patients with chronic HCV infection, and in such patients, this occult infection may be associated with more severe liver damage and even the development of HCC [14]. Occult HBV infection has found in individuals without HBV serological markers, past HBV infection, and with HCC patients with or without chronic hepatitis C [15]. Occult HBV infection has been associated with cryptogenic chronic hepatitis and HCC. Furthermore, some studies suggested that occult hepatitis B might affect responsiveness of chronic hepatitis C to interferon therapy and disease progress [14].

There is little information of HBV genotypes and its relation to occult infection despite the importance of this infection in Egypt. Taking into consideration the impact of the fact that HBV and HCV infections are common in Egyptian HCC patients, we investigate the genotyping of occult HBV infection in Egyptian HCC patients with or without HCV infection.

## Methods

This study included 40 histological confirmed HCC-HBsAg-negative Patients; with mean age of 55 ± 4.9 years, male to female ratio 4:1, who underwent surgical resection in the National Cancer Institute-Cairo University. Ten patients had HBsAg-positive samples and included as a control group, tissue and blood samples were obtained from all the patients. The study protocol was performed according to the principles of Helsinki Declaration, and informed consent was obtained from all the patients.

All serologic markers for HBV infection (HBsAg, anti-HBs, anti-HBc, and anti-HBs) were detected with current standard assays (Adalts, Italy) and antibodies to HCV with HCV EIA version 3.0 according to the manufacturer's instructions (Adalts, Italy).

### Detection of occult HBV DNA

In this study, we investigated the presence of HBV DNA by nested-PCR. We use three primer sets each specific for Surface, Core, and X viral genomic regions, respectively, in both tumor liver tissues and sera of patients as previously described [16] (Table 1). DNA extracted from the serum by QIAGEN viral DNA extraction kit (QIAGEN) using 140 μl of patient serum according to the manufacturer's procedure, and from frozen liver specimens by standard Procedures [16]. In brief, 50 mg tissue specimens were homogenized in buffer (150 mmol/L NaCl, 50 mmol/L Tris-HCl (pH7.4), 10 mmol/L EDTA, 1% SDS), and incubated overnight with proteinase K (800 μg/mL) at 37°C. After extraction with phenol/chloroform the nucleic acids were precipitated with ethanol. Nucleic acids were then resuspended and digested with pancreatic ribonuclease (100 μg/mL) followed by extraction with phenol/chloroform, and re-precipitation in pure cold ethanol. The DNA was resuspended in 10 mmol/L Tris-HCl (pH 7.4), 1 mmol/L EDTA, and its concentration was determined by using a UV spectrophotometer (Nanodrop). Amplification of the β-actin gene was performed to test for the presence of artifacts as well as to set a baseline for tissue sample that enables the evaluation of the target genes in the HBV PCR. The amplified products were visualized on an ethidium bromide-stained 2% agarose gel.

**Table 1:** Sequences of primer pairs^16 ^used for HBV PCR detection:

Core gene		
C1s:	5'CTGGGAGGAGTTGGGGGA3'	1730-1747
C2a:	5'GTAGAAGAATAAAGCCC3'	2503-2487
C3s:	5'GGTCTTTGTACTCGGAGGCTG3'	1763-1783
C4a:	5'ATACTAACATTGACATTCCC3'	2455-2436
Surface gene		
S-1s:	5'AGAACATCGCATCAGGACTC3'	159-178
S-2a:	5'CATAGGTATCTTGCGAAAGC3'	642-623
S-3s:	5'AGGACCCCTGCTCGTGTTAC3'	181-200
S-4a:	5'AGATGATGGGATGGGAATAC3'	619-600

X gene		
X1s:	5'CTAGCCGCTTGTTTTGCTCG3'	1282-1301
X2a:	5'TTATGCCTACAGCCTCCTAG3'	1666-1647
X3s:	5'GGTCTTACATAAGAGGACTC3'	1518-1537
X4a:	5'GTTCACGGTGGTCTCCAT3'	1625-160

### HBV-Genotype analysis

The determination of genotypes A through F of hepatitis B virus was done according to previous described method [17,18]. The sequences of PCR primers used in this study are shown in table 2. In brief, the first and second- round PCR primers were designed on the basis of the conserved nature of nucleotide sequences in regions of the pre-S1 through S genes, irrespective of the six HBV genotypes [17]. P1 and S1-2 were universal outer primers. B2 was used as the inner primer with a combination called mix A for genotypes A, B, and C. The first PCR was carried out in 40 ul of a reaction mixture containing 100 ng of each outer primer, a 200 mM concentration of each of the four deoxynucleotides, 2.5 U of Taq DNA polymerase (Promega, France) 1× PCR buffer containing containing (50 mM KCl, 10 mM Tris pH 8.3) and 1.5 mM MgCl2. The cycling protocol included one cycle of 5 min at 95°C, followed by 40 cycles consisting of 94°C for 1 min, 55°C for 1 min and 72°C for 2 min. Two second-round PCRs were performed for each sample, with the common universal sense primer (B2) and mix A for types A through C and the common universal antisense primer (B2R) and mix B for types D through F.

**Table 2:** HBV genotyping primers sequence

Primer Sequence	*a*(position, specificity, and polarity)
**First PCR**	
P1 5'-TCA CCA TAT TCT TGG GAA CAA GA-3'	(nt 2823-2845, universal, sense)
S1-2 5'-CGA ACC ACT GAA CAA ATG GC-3'	(nt 685-704, universal, antisense)
**Second PCR**	
**Mix A**	
B2 5'-GGC TCM AGT TCM GGA ACA GT-3'	(nt 67-86, types A to E specific, sense)
BA1R 5'-CTC GCG GAG ATT GAC GAG ATG T-3'	(nt 113-134, type A specific, antisense)
B1R 5'-CAG GTT GGT GAG TGA CTG GAG A-3'	(nt 324-345, type B specific, antisense)
BC1R 5'-GGT CCT AGG AAT CCT GAT GTT G-3'	(nt 165-186, type C specific, antisense)
**Mix B**	
BD1 5'-GCC AAC AAG GTA GGA GCT-3'	(nt 2979-2996, type D specific, sense)
BE1 5'-CAC CAG AAA TCC AGA TTG GGA CCA-3'	(nt 2955-2978, type E specific, sense)
BF1 5'-GYT ACG GTC CAG GGT TAC CA-3'	(nt 3032-3051, type F specific, sense)
B2R 5'-GGA GGC GGA TYT GCT GGC AA-3'	(nt 3078-3097, types D to F specific, antisense)

*a *An "M" represents a nucleotide that could be either an A or a C; a"Y" represents a nucleotide that could be either a C or a T. nt: nucleotide.

A 1 ul aliquot of the first PCR product was added to two tubes containing the second sets of each of the inner primer pairs, each of the deoxynucleotides, Taq DNA polymerase, and PCR buffer, as in the first reaction. These were amplified for 40 cycles with the following parameters: preheating at 95°C for 5 min, 30 cycles of amplification at 94°C for 1 min, 58°C for 1 min, and 72°C for 1.30 min Genotypes of HBV for each sample were determined by identifying the genotype-specific DNA bands. The two different second-round PCR products from one sample were visualized on an ethidium bromide- stained 3% agarose gel.

### Detection of HCV RNA

Nucleic acid extraction was done by Mini-extraction kit (QIAGEN) using 140 μl of patient serum according to manufacturer's procedure. RT and PCR were done as previously described [17] with primers from the most conserved 5' UTR of viral genome.

### Statistical analysis

Data were analyzed by the chi-square test. A *P *value of less than 0.05 considered statistically significant.

## Results

Characteristics of the participants were shown in table 3. Out of these forty patients, 19 had serological positive HCV infection with elevated ALT. Tissue and serum samples were considered HBV DNA reactive if at least two of the three PCR assays positive. The intrahepatic HBV genes were the Surface 18/40 (45%), X 16/40 (40%) and Core genes 26/40 (65%) in the occult patients compared to 10/10(100%), 6/10(60%) and 6/10(60%) in control group respectively. There was no difference observed in prevalence of intrahepatic occult HBV infection in relation to age or sex.

**Table 3:** The characteristics of the study subjects

Characteristics	
No. of the participants	40
No. of the controls	10
Age-years (range)*	55 ± 9 (25-72)
Sex Male/Female	32/8
Elevated serum ALT levels with HCV Ab	19/40 (45%)
Anti-HBs	16/40(40%)
Anti-HBc	30/40(75%)

*Expressed as mean+SD (range); ALT (normal range (0-41 IU/L)

In the HBsAg negative HCC patients, out of 25 intrahepatic HBV-DNA, 40% had HCV-RNA, 72% had liver cirrhosis. According to tumor grade 16%, 52% and 32% had tumor grade I, II, and III respectively. Intrahepatic HBV DNA was detected in (25/40) 63%, of them seven (28%) had both X and Core genes, five (20%) had both S and X genes, and 11(44%) had S and C genes. Only two cases out of 25 (8%) had all genes. In the control group, HBV-DNA was detected in all liver tissues, all had S-gene, of them six had both X and Core genes. Out of the twenty-five cases eleven (44%) had both anti-HBc-IgG and anti-HBs. HCV RNA was found in the serum of 10/25 (40%). Nine of ten (90%) HCV-RNA positive patients had detectable serum HBV-DNA. Seventy-two percentage of these cases (18/25) had liver cirrhosis. HBV DNA-positive patients had significantly high tumor grade I and II compared to HBV-DNA negative patients.

In serum, HBV-DNA was detected in 20 of 40 patients (50%), 16/40 (40%), 13/40 (32.5%), 4/40 (10%) had X, S and Core gene respectively. HBV DNA were detected in only 9/40 (22.5%) of HCC patients. Among the 40 HCC patients with different serological patterns for HBV infection, occult HBV infection was 4/40 (10%) in cases positive for both anti-HBs and anti-HBc. The overall prevalence was significantly higher for those positive for anti-HBc alone or with anti-HBs than for those negative for all markers (p = 0.001).

This study showed, as in table 4, that intrahepatic HBV infections are attributed predominantly to viral genotypes D and B that constituted 8/25 (32%) and 6/25 (24%), respectively. HBV genotypes A and C infections were the least observed and constituted 4% and 8% respectively compared 100% of HBV genotype A and D mixed infections in the control group with no other genotype observed. In addition, there was a relatively high prevalence of mixed infections of 5/25(20%) among the studied group. One case had both genotypes A and D, 2 cases had both B and D and 2 cases had genotypes B and C. No HBV genotype E or F was found in our study and furthermore, genotypes G and H were not determined.

**Table 4:** Correlation between clinical and virological data in the intrahepatic HBV DNA

Variables	No.(%)
HCV-RNA	10/25 (40%)
High AFP	15/25(60%)
Liver cirrhosis	18/25(72%)
Tumor Grade I	4/25(16%)
Tumor Grade II	13/25(52%)
Tumor Grade III	8/25(32%)
X-gene and HBcIgG +ve	12/25(48%)
S-gene and HBcIgG +ve	16/25(64%)
C-gene and HBcIgG +ve	20/25(80%)
HBV genotype A	1/25 (4%)
HBV genotype B	6/25 (24%)
HBV genotype C	2/25 (8%)
HBV genotype D	8/25 (32%)
HBV mixed genotype	5/25 (20%)

In the serological HCV-related HCC, the serum occult HBV infection was 47.4% (9/19). The prevalence of occult HBV infection was higher in patients having both anti-HBs and anti-HBc 2/4 (50%) than those having anti-HBc alone 1/5 (20%). In our previous study among those 25 patients with occult HBV infections, HCV genotypes 4a was the most common [19], and according to the presence or absence of occult HBV infection, there was no significant difference in the mean serum ALT level.

## Discussion

Occult HBV infection is characterized by positivity for HBV DNA in HBsAg-negative patients with or without serological markers of previous HBV infection [20]. Occult HBV infection has been commonly reported among immunocompromised patients [21-23]. In Egypt, occult HBV infection is 9/712 (1.26%) in the blood donors accepted blood donations [24]. In this study, the prevalence of the intrahepatic HBV-DNA was 63%. Similarly, in another studies on the HCC patients, it varies from 22%- 87% [12,25] and among general population in different countries to be ranged from 2-16% [15,26]. Occult HBV prevalence was significantly high among HCC, chronic hepatitis C, or under hemodialysis [10,27,28]. HBV may be also reactivated in patients undergoing anti-cancer chemotherapy [12,29]. Occult HBV infection may occur after complete clinical recovery from acute self-limited hepatitis so; HBsAg seroclearance does not necessarily imply HBV eradication [30,31]. In endemic areas, the anti-HBc IgG and anti-HBs as well as HBsAg were not sufficient markers to exclude HBV-DNA carriers [32].

The presence of occult HBV during chronic HCV infection is well described, the observed high proportion of HCV-related HCC cases show occult HBV infection, was similarly in the previous epidemiological surveys that found close relationship between the existence of HCV and the occurrence of HCC in HBsAg-negative HCC [20,33,34]. Our study showed the serum occult HBV infection to be 47.4% (9/19) in HCV- HCC patients. The prevalence of occult HBV infection was particularly high among patients with HBV antibodies.

In our study, the intrahepatic HBV DNA was detected in 42.1% (8/19) of the HCV-Ab positive patients. Only 14.2% (3/21) patients without HCV-Ab have HBV-DNA in the liver tissue. Similarly, its prevalence in HCV patients is reported to be 40-50% in liver tissue [7,9]. There was an elevated risk in HCV patients because this silent infection can affect the progression and HCC development [19].

The prevalence of occult HBV infection was higher in subjects having either anti-HBs or anti-HBc or both anti- HBs and anti-HBc. Our results revealed that in 19 patients with HCV infection, it was higher in patients having both anti-HBs and anti-HBc 50% than those having anti-HBc alone 20%. In an Egyptian chronic HCV patients study, they found that those positive for anti-HBc had severe liver disease compared to negative ones. Serum HBV-DNA was 22.5% in anti-HBc-positive chronic HCV patients and nothing detected in anti-HBc-negative chronic HCV patients [35]. Similarly, serological findings in patients with occult hepatitis B and HCV co-infection revealed that 35% of people were HBsAb positive, 42% were anti-HBc IgG positive, and 22% were negative for both [28]. In Northern countries, no more than 5% of HBsAg-/anti-HBc+ blood donor samples contain HBV DNA [36]. In West Africa, 5% of total HBV DNA carriers are HBsAg negative [37].

HCC patients living in endemic areas for HBV were frequently found positive for HBsAg and/or anti-HBc antibodies and this strong relationship was the first epidemiological evidence of HBV-related oncogenic transformation [38]. Among our 40 HCC patients with different serological patterns for HBV infection, the prevalence of occult HBV infection was 10% in both anti-HBs and anti-HBc positive cases, and 0% in cases negative for all markers. The overall prevalence was significantly higher for those positive for anti-HBc alone or with anti-HBs than for those negative for all markers. Data presented here sowed that out of the intrahepatic HBV DNA positive cases, 56% were only anti-HBc-IgG positive, while 44% were both anti-HBc-IgG and anti-HBs positive. A higher frequency of HBV-DNA, among anti-HBc positive patients 46% than in anti-HBc negative patients (20%) has previously reported [39]. We suggested that while HBV is not the only factor in HCC development in Egypt, it is still one of the major risks.

Occult HBV has associated with more advanced fibrosis/cirrhosis [40]. Our study shows that 72% of the HCC patients with intrahepatic occult HBV had cirrhosis. Cirrhosis is considered as an important risk factor for the development of HCC [41-44]. In addition, occult HBV infection may favor neoplastic transformation in HCV-infected patients through its contribution to cirrhosis. Many epidemiologic and molecular studies indicate that persistent HBV infection may have a critical role in the development of HCC in HBsAg-negative patients [45].

Using PCR primers located in several HBV genes, HBV DNA can be amplified in many tissues but not all cases [46]. In this study, intra-hepatic HBV genes were examined in the forty HCC participants in whom the Surface, X and Core genes were detected in 45%, 40%, and 65%, respectively. In intra-hepatic HBV DNA positive cases, 28% were positive for X and Core genes, 20% were positive for Surface and X genes, and 44% were positive for Surface and Core genes. Only 8% were positive for Surface, X and Core genes. The prevalence of occult HBV infection did not differ with age or sex. The persistent HBV infection may have a critical role in the development of HCC in HBsAg-negative patients. One of the markers in HCC cases, HBsAg (-), has been the presence of the HBV-X gene expression in HCC since positivity for the HBV-X protein in liver tissue in several studies reached half of the liver tissues specimens. In several reports the PCR for S gene was sensitive in serum whereas the X gene was sensitive in the liver [28]. The X gene deregulates cell cycle control, interferes with cellular DNA repair and apoptosis, and plays an important role in interaction with p53 and Rb gene [47].

HBV genotype was a factor that influencing the frequency of occults HBV. In this study the intrahepatic HBV infections in HCC cancer patients are predominantly to viral genotypes D and B that constituted 8/25 (32%) and 6/25 (24%), respectively. HBV genotypes A and C infections were the least observed and constituted 4% and 8% respectively. In addition, there was a relatively high prevalence of mixed infections of 5/25(20%) among the studied group. One case had both genotypes A and D, 2 cases had B and D and 2 cases had genotypes B and C. Similarly, in our previous study, we found that HBV mixed genotype infections could probably be of clinical significance in HBV-induced liver diseases, in which a prevalence of mixed genotype infections in the study participants was 15.7% especially those with CAH. HBV genotype A and D mixed infections accounted for 45.5% of the total mixed infections. Occult HBV during the non-replicative phase is therefore expected to be more frequent in areas where genotypes A, D, and E are prevalent [18].

In conclusion, occult HBV infection and the HBV genotype B or D may influence the outcome of HBV infection leading to the development of hepatocellular carcinoma and may have a strong association with HCV in the carcinogenesis of liver. A decrease in the immune status may result in HBV reactivation in anti-HBs positive patients undergoing chemotherapy.

## Conflict of interests

The authors declare that they have no competing interests.

## Authors' contributions

ZKH: Conceived of the study, participated in its design and coordination, sample collection and carried out the serological and molecular assays, and drafted the manuscript. MMH: participated in the study design, sample collection, carried out the molecular genotyping studies, participated in the drafted the manuscript and performed the statistical analysis and the manuscript submission. ARNZ: participated in the study design and in the editing of the manuscript. TMM: Coordinated the research effort. All authors read and approved the final manuscript.
